# Androgens in women: Establishing reference intervals for dehydroepiandrostenedione sulphate and androstenedione on the Roche Cobas

**DOI:** 10.11613/BM.2023.020706

**Published:** 2023-06-15

**Authors:** Adriana Bokulić, Ivana Zec, Domagoj Marijančević, Sanja Goreta

**Affiliations:** Department of Clinical Chemistry, Sestre Milosrdnice University Hospital Center, Zagreb, Croatia

**Keywords:** reference intervals, androgens, dehydroepiandrostenedione sulphate, androstenedione, immunoassay, women

## Abstract

**Introduction:**

Immunoassays are the most common method in routine practice for measuring androgens in women. Study’s aim was to establish new population specific indirect reference intervals (RI) for dehydroepiandrostenedione sulphate (DHEAS) and for new androstenedione test available on automated Roche Cobas electrochemiluminescent immunoassay method.

**Materials and methods:**

From extracted laboratory records, testosterone, sex hormone binding globulin and follicle-stimulating hormone were used as reference tests to exclude possibly diseased women. After the data selection steps, the study included 3500 subjects for DHEAS and 520 for androstenedione aged 20-45 years. To evaluate the need for age partitioning, we calculated standard deviation ratio and bias ratio. For each hormone, 90% and 95% RIs were calculated with appropriate statistical method.

**Results:**

Total age group (20-45 years) 95% RIs were: 2.77-11.50 µmol/L for DHEAS and 2.48-8.89 nmol/L for androstenedione. Age-stratified 95% RIs for DHEAS were: 3.65-12.76 µmol/L (20-25 years); 2.97-11.50 µmol/L (25-35 years) and 2.30-9.83 µmol/L (35-45 years). Age-stratified 95% RIs for androstenedione were: 3.02-9.43 nmol/L (20-30 years) and 2.23-7.75 nmol/L (30-45 years).

**Conclusion:**

New RIs for DHEAS were slightly wider for age group 20-25 and 35-45, while the differences in the age group 25-35 years were more pronounced. Androstenedione RI showed significantly higher concentrations than the manufacturer’s. Age-related decrease of androgens should be considered when calculating RIs. We propose population specific, age-stratified RIs for DHEAS and androstenedione on electrochemiluminescent method, which should improve test interpretation in women of reproductive age.

## Introduction

Androgens have significant importance in women’s reproductive health as they play a key role in normal ovarian function and fertility. Assessment of androgen production in women usually includes testosterone, dehydroepiandrostenedione sulphate (DHEAS) and androstenedione. In addition, sex hormone binding globulin (SHBG) is measured to calculate free testosterone or free androgen index (*1*, *2*). Androgens are usually measured in assessment of polycystic ovary syndrome (PCOS), but various other conditions are linked to altered concentration as osteoporosis, sarcopenia, mental disorders, cardiovascular diseases, memory loss and loss of sexual desire. Their concentration is also changed with the use of hormone replacement therapy (HRT) and oral contraceptive pills (OCP) (*3*). Although testosterone is the main androgen, DHEAS and androstenedione are often also requested as part of initial hormone assessment.

Steroid measurement remains a challenge for the healthcare community, especially in terms of poor analytical sensitivity at low concentrations in females. All the direct androgen immunoassays suffer from poor analytical sensitivity and lack of specificity due to cross-reactivity and matrix interferences. Poor standardization data is causing variability among different kits and laboratories and consequently leading to significant variations between corresponding reference intervals (*4*). Since immunoassays are still widely used, method specific reference interval (RIs) are of the utmost importance. Liquid chromatography-tandem mass spectrometry methods (LC-MS/MS) proved to be sensitive method for measuring low female steroid concentrations, but often unavailable in routine practice. Due to LC-MS/MS specificity, the reference ranges for androgens may be somewhat lower than those by immunoassays (*5*, *6*).

As production declines from reproductive age towards menopause, there is a need for age specific RIs for more precise interpretation of androgen excess or insufficiency (*7*, *8*). Reference intervals are the most common decision support tool used by healthcare to interpret numerical laboratory test results, ideally allowing distinction of healthy and unhealthy individuals. In general, laboratories are responsible for either verifying RIs established by an external source or determining their own (*9*). The traditional direct approach for establishing RIs is well defined in CLSI EP28-A3c guidelines (*10*). It is based on collecting samples from the preselected reference population, followed by analysis and calculation. The main obstacle lies in selecting a sufficient number (> 120) of healthy subjects for reference population for each subgroup (for example by age or sex). Alternatively, there is an increased demand for indirect methods of establishing RIs using routine laboratory data stored in the laboratory information system (LIS) with appropriate statistical techniques. The biggest advantages over direct approach are that it is faster, cheaper and more convenient. Key disadvantages of indirect approach can be found in sample filtering and method of calculation (*11*). When selecting subjects, the main premise is not to include only healthy subjects, rather to minimize the effect of diseased subjects. The steps how to filter out diseased subject are not standardized. Multiple statistical techniques are available for calculating the interval, such as standard parametric and non-parametric statistics, Bhattacharya method and Kolmogorov-Smirnov distance (*i.e*. kosmic algorithm) (*12*-*14*). The choice of method often depends on user’s understanding of the principle and the availability of software tools for calculation.

There is lack of literature data concerning RIs for DHEAS and androstenedione on electrochemiluminescent immunoassay (ECLIA). In our case, direct approach would require large number of women in whom we would have excluded at least ovary and adrenal gland diseases. We opted for indirect calculation, as it did not require such extensive work. This study aims to establish new indirect RIs on our population of women of reproductive age and to compare our results to manufacturer-derived RIs.

## Materials and methods

### Subjects

The study was carried out on in- and out- patients visiting the Laboratory of Endocrinology of the Sestre Milosrdnice University Hospital Centre, Zagreb, Croatia after approval by ethics review board. Almost 16,000 records have been extracted from LIS over period of 7 years (February 2014 to October 2021) for women aged 20 to 50 years containing concentration of any of the androgens: testosterone, DHEAS and androstenedione. Additionally, extracted data included concentration of SHBG and follicle-stimulating hormone (FSH) if available.

### Sample collection and assays

Samples were collected in fasting state from 7.00 to 10.00 am by venepuncture in test tubes with clot activator (4 or 7 mL Vacuette, Greiner Bio-One GmbH, Kremsmünster, Austria). After clotting, blood samples were centrifuged at 2200xg for 10 minutes and fresh serum samples were used for analysis. All measurements, except androstenedione, were performed on Roche Cobas analysers e601 until March 2020 and e801 from April 2020 (Roche Diagnostics GmbH, Mannheim, Germany) by ECLIA method. Two different generations of Roche analysers (e601 and e801) were compared in our laboratory using at least 20 patient samples with values covering all measuring range. The comparison results showed no clinically significant difference (Supplement 1). Androstenedione was measured only with Roche Cobas e801 ECLIA method and implemented in routine practice in October 2020. All calibrations, internal and external quality control assessment were carried out according to the laboratory-defined standard operating procedures throughout all study duration.

### Data selection

**Figure 1:**
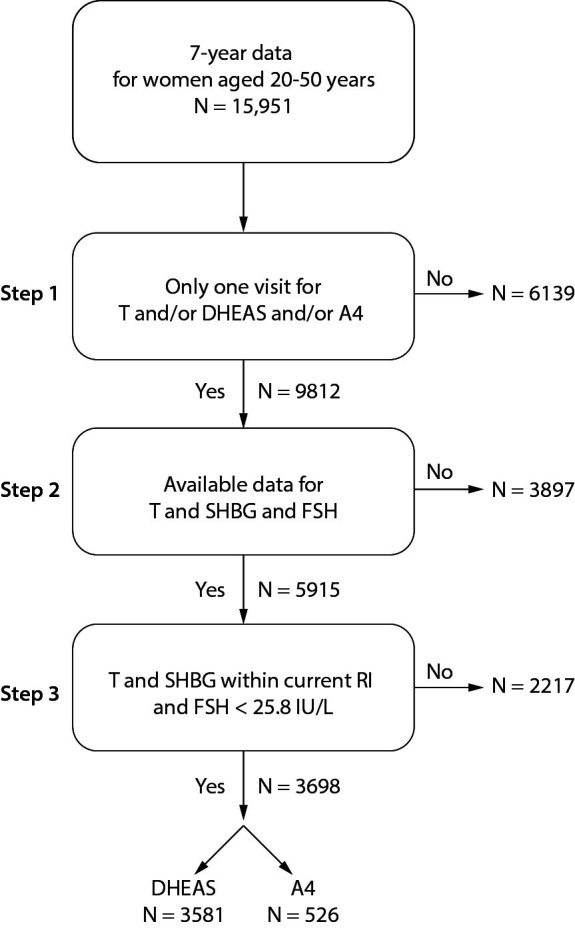
Flow chart of data selection. T - testosterone. DHEAS – dehydroepiandrostenedione sulphate. A4 - androstenedione. SHBG - sex hormone binding globulin. FSH - follicle-stimulating hormone. RI - reference interval.

Data selection is shown in Figure 1. From the initial 15,951 records, we eliminated all records from patients with more than one visit in extracted timeframe of 7 years. Rationale was that patients with repeated testing of any androgen had higher chance of having endocrine disorder. From this patient unique dataset, we included only records containing all three reference tests (testosterone, SHBG and FSH). In the last step, we applied exclusion criteria based on whether concentration of reference tests falls within manufacturer defined RIs (Table 1). Testosterone is considered primary and the most important androgen and patients with normal testosterone concentration are considered to have lower probability of hyperandrogenemia. Concentration of SHBG is altered in OCP use or PCOS, which both influence androgen production. FSH concentration less than 25.8 IU/L was used to exclude menopause (manufacturer-defined lower RI for postmenopause). DHEAS or androstenedione was considered eligible for new RI if: (i) both concentrations of testosterone and SHBG were within current RIs and (ii) FSH concentration was less than 25.8 IU/L.

**Table 1:** Reference interval derived from manufacturer’s instructions for use (90%RI)

	

			**Percentile**		
**Analyte (unit)**	**Age, years**	**N**	**5^th^**	**50^th^**	**95^th^**
Testosterone (nmol/L)	20-50	89	0.29	0.94	1.67
SHBG (nmol/L)	20-50	166	32	68	128
	20-25	36	4.02	6.46	11.0
DHEAS (µmol/L)	25-35	64	2.68	4.96	9.23
	35-45	85	1.65	4.38	9.15
	45-55	89	0.96	3.28	6.95
Androstenedione (nmol/L)	20-50	84	1.71	2.89	4.59
SHBG - sex hormone binding globulin. DHEAS – dehydroepiandrostenedione sulphate.					

### Statistical analysis

Data analysis were performed according to CLSI EP28-A3c guidelines (*10*). Because of large number of samples, data for visual inspection and results were grouped in 5-year periods. Whole purified data was plotted as histogram to see frequency of age and sample origin. For each hormone, data was plotted as Box-Cox diagram against age and visually inspected for possible stratification. Shapiro-Wilk test was applied to test normality. Outliers were tested using Tukey method after Box-Cox transformation and subsequently eliminated. For each group, 90% and 95% RIs were calculated with appropriate statistical method as follows: (i) parametric with normal distribution after Box-Cox transformation; (ii) nonparametric percentile for non-Gaussian distribution. There is no single recommendation how to calculate indirect RIs once reference population is defined. Different calculation methods can give different limits (*15*). We opted for standard CLSI based calculation after exclusion of outliers by Tukey, but other approaches can be used (*12*, *13*). To evaluate the need for age partitioning, we used standard deviation ratio (SDR) and bias ratio (BR) as explained by Omuse *et al.* (*16*). Standard deviation ratio indicates differences between age groups at the centre of RI distribution while BR reflects differences in upper or lower limit of RI between groups. If SDR was above 0.4 and bias ratio above 0.375 for lower or upper limit of RI, we considered age partitioning appropriate and justifiable. Results were provided through statistical software MedCalc version 19.2.1 (MedCalc Software Ltd, Ostend, Belgium) and Minitab version 19.2 (Minitab Statistical Software, AppOnFly Inc., San Francisco, USA).

## Results

**Figure 2:**
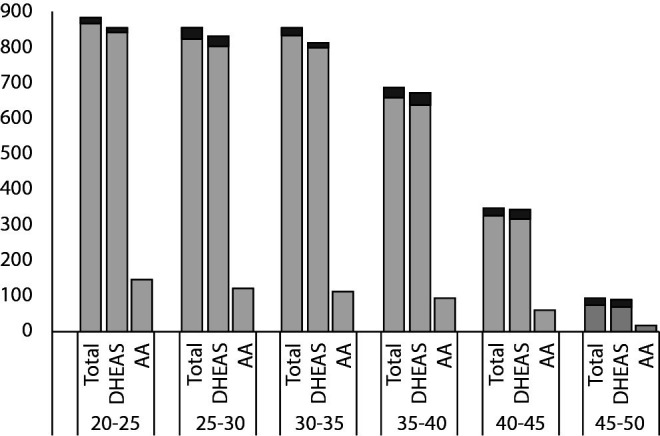
Number of subjects (N = 3698) for total number and separately for dehydroepiandrostenedione sulphate (DHEAS) and androstenedione (A4) according to age groups with in-patients shown in dark and out-patients in light grey colour.

**Figure 3:**
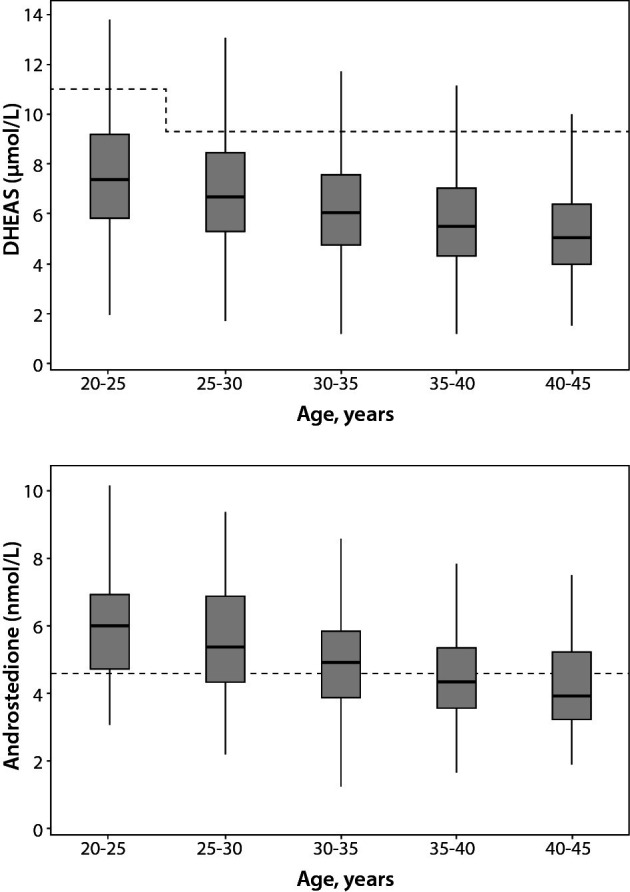
Box-plot diagram of each hormone plotted against 5-year age groups. Dashed lines indicate upper limit of current reference interval defined by Roche. The box-plot shows 25^th^, 50^th^ (median) and 75^th^ percentile, while whiskers show range without outliers. DHEAS – dehydroepiandrostenedione sulphate.

Flow chart for establishing new RIs with the previously described three steps of data elimination and selection is shown in Figure 1. Because of repeated visits and measurements of the same analytes, 12,156 patients produced 15,951 records. Figure 2 shows data after applying all three steps of elimination as histogram of frequency of age and sample origin. The largest group of patients is the youngest group aged 20-25 years, while the oldest group of women aged 45-50 included only 85 records. Histogram also shows that majority of data is from outpatients, ranging from 86% (45-50 years) to 99% (20-25 years). On this set of 3698 records, calculated median, 25^th^ and 75^th^ percentiles for reference tests were as follows: testosterone 1.0 (0.8-1.3) nmol/L, SHBG 65 (50-85) nmol/L and FSH 6.4 (5.3-7.7) IU/L. Since the oldest group (45-50 years) included only 85 patients (81 results for DHEAS and 6 for androstenedione), we additionally excluded them from calculation of RIs as it was considered too small number to represent this age-group. Finally, number of samples for each analyte was reduced to 3500 for DHEAS and 520 for androstenedione and upper age limit was reduced from 50 to 45 years. Figure 3 shows this final data as box-plot diagram plotted against age. Reference intervals were calculated with total age group (20-45 years) and with subgroups based on our visual inspection. Outliers were tested for each group separately and subsequently eliminated. Reference intervals are shown in Table 2, both as 90% and 95% range, and both as total and age-stratified for easier comparison of our results with Roche (Table 1) and other studies. Calculated SDR for DHEAS was 0.43, BRs for lower and upper limit were 0.300 and 0.566 (95%RI 20-25 *vs* 95%RI 25-35 years) and 0.306 and 0.750 (95%RI 25-35 *vs* 95%RI 35-45 years). For androstenedione, calculated SDR was 0.54, BRs for lower and upper limit were 0.483 and 1.028 (95%RI 20-30 *vs* 95%RI 30-45 years).

**Table 2:** Study’s reference interval defined as percentiles

	**Age, years**				**Percentile**			
**Analyte (unit)**	**total**	**stratified**	**N**	**2.5^th^**	**5^th^**	**50^th^**	**95^th^**	**97.5^th^**
	20-45*		3455	2.77	3.22	6.31	10.74	11.50
DHEAS (µmol/L)		20-25^†^	842	3.65	4.16	7.4	11.79	12.76
		25-35*	1627	2.97	3.32	6.37	10.64	11.50
		35-45^†^	990	2.30	2.71	5.41	9.03	9.83
	20-45^†^		518	2.48	2.83	5.13	8.20	8.89
Androstenedione (nmol/L)		20-30^†^	267	3.02	3.38	5.76	8.75	9.43
		30-45^†^	250	2.23	2.54	4.51	7.16	7.75
DHEAS and androstenedione are calculated as total reference interval (RI) and with stratified age groups. RIs are defined with 5^th^ and 95^th^ percentile (90%RIs) and with 2.5^th^ and 97.5^th^ percentile (95%RIs). Median is 50^th^ percentile. *Non-parametric percentile method. ^†^Parametric method after Box-Cox transformation. DHEAS – dehydroepiandrostenedione sulphate.								

## Discussion

This study establishes both 90 and 95% indirect RIs for DHEAS and androstenedione in women of reproductive age using ECLIA method. Literature data regarding androgens in women by ECLIA method is scarce and our results can help androgen results interpretation on population specific to our laboratory.

Median values of our final selected data showed good agreement with the ones declared by the manufacturer: testosterone (1.0 *vs* 0.94 nmol/L) and SHBG of (65 *vs* 68 nmol/L), giving us assurance that data cleaning was successful. Similar medians can be found in other studies establishing RIs for testosterone and SHBG for ECLIA method (*17*-*19*).

Our study results show well-known age-related decrease of androgens. Data stratification is not only justified with physiological changes but also confirmed by calculating the SDR and BR. Based on SDR, DHEAS shows minimal difference in centre of distribution between subgroups. Bias ratio shows significant difference only in the upper limit, which is often expected in skewed distributions. For androstenedione, significant difference was found in both criteria, SDR and BR. We can conclude that age partitioning is justifiable for both hormones. Other studies, regardless of method used, also established RIs by age (*20*-*23*).

The majority of our subjects were younger than 35 years of age as androgens are more often tested in younger women evaluating fertility. However, unequal age distribution can cause bias when calculating reference limits (*15*). Balanced age distribution for DHEAS is noted in the first two groups of RIs (20-25 and 25-35 years group). Age group 35-45 years consists of 990 subjects but with twice the number of subjects within 35-40 than 40-45-year group. The similar pattern of age distribution can be seen in androstenedione, with the first stratified age group (20-30 years) having equal number of subjects while older (30-45 years) showing decline in numbers with age. The eldest group (45-50 years) was excluded from calculation for both hormones due to extremely small number of participants. Merging this small dataset with the former group is also not possible as it could cause significant age bias.

Immunoassays usually overestimate androgen concentration in women compared to mass spectrometry and Roche is no exception, as demonstrated for DHEAS by Büttler *et al.* (*24*). Our external quality control results for androstenedione (supplied by The United Kingdom National External Quality Assessment Service) showed good correlation to mass spectrometry, giving higher bias (24.7%) at lower concentration and smaller bias (10.3%) at normal to high concentration. Only two studies compared ECLIA to LC-MS/MS; both Obermayer-Pietsch *et al*. and Wei *et al*. showed excellent agreement between these two methods (*25*, *26*). Based on previously mentioned studies and our external quality control results, androstenedione RIs established by ECLIA method should be in very good agreement to mass spectrometry.

Calculated 90% RIs for DHEAS show slightly positive shift in both sides of the distribution for groups 20-25 and 35-45 years compared to age-matched 90% RIs from the manufacturer. Although overall they are in good agreement, we have significantly higher medians. Different sample number could cause this discrepancy. Larger difference, especially in the upper limit, is seen only in age partition 25-35 years (10.64 *vs* 9.23 µmol/L). Surprisingly, there are no other method-matched study. Few other studies are defining RIs using LC-MS/MS, but due to different age partitioning, we can only compare total RIs. As expected, our ECLIA results are significantly higher compared to LC-MS/MS studies (*20*, *21*).

Median for our androstenedione total RI was higher than the manufacturer-declared upper reference limit (5.13 *vs* 4.59 nmol/L), meaning that more than half of our subjects had high androstenedione concentration (Figure 3). Our calculated upper reference limit was almost twice as high (8.20 *vs* 4.59 nmol/L). Differences in the lower limit are less pronounced, calculated 2.83 *vs* declared 1.71 nmol/L, but still present. This is highly surprising as both RIs are defined on similar European population. Even though our number of samples for indirect method is not large, differences between medians and upper reference limits are too big to be ignored. Wei *et al*. published the only other method-matched study on American patients, giving 97.5^th^ percentile significantly lower than ours (5.6 *vs* 8.89 nmol/L) (*26*). Possible reason for this is population differences and study design. There are several publications defining RIs using mass spectrometry. Our total and age-stratified results are expectedly, slightly higher than LC-MS/MS, but still in good agreement (*21*-*23*). In contrast, Fanelli *et al*. showed results more similar to manufacturer-stated RI than ours (95%RI 1.0-5.7 nmol/L) (*27*). Considering well-known positive bias of immunoassays and population similarities, we cannot explain observed discrepancy of Roche-declared RIs to both ours and most LC-MS/MS studies. Direct RI for androstenedione by Roche ECLIA method on our population should be calculated in additional study to confirm this major discordance.

Several limitations of this study should be noted. Our exclusion criteria removed only part of possibly diseased subjects. We did not take into account OCP or menstrual cycle irregularities which could influence androgen concentration (*20*, *21*, *28*). We did not record phase of menstrual cycle. According to some studies, phase of menstrual cycle influences androgen concentration, while other studies found no such difference (*18*, *24*, *26*). Our data showed uneven age distribution in the oldest groups (35-45 years for DHEAS and 30-45 years for androstenedione), which can influence calculated RIs. There is option to randomly delete data in oversized age group in order to reduce between year variations (*15*). We decided not to delete data assuming that influence of the skewed age distribution is preferable to downsizing amount of data. This decision could possibly lead to slightly higher calculated RIs for those age groups. For indirect RIs, the rule for the number of data can be summarized as the bigger, the better. Recommended number of samples (> 400) was not reached for age-stratified androstenedione RIs (*12*).

This study shows significant differences in calculated *vs* manufacturer-reported RIs. This confirms laboratories should verify manufacturer-reported RIs before implementing them in routine practice, especially when new test becomes available on the market. Our data shows that age partitioning of androgens in women is justifiable. Study presents new indirect RIs for DHEAS and androstenedione on ECLIA method in two ways: (i) 90 and 95% RIs; (ii) total and age-stratified. We have implemented 95% age-stratified RIs derived from this study, and we encourage other laboratories to do the same.

## References

[1] Davison SL Bell R. Androgen Physiology. Semin Reprod Med. 2006;24:71–7. 10.1055/s-2006-939565 16633980

[2] Astapova O Minor BMN Hammes SR. Physiological and Pathological Androgen Actions in the Ovary. Endocrinology. 2019;160:1166–74. 10.1210/en.2019-00101 30912811 PMC6937455

[3] Bianchi VE Bresciani E Meanti R Rizzi L Omeljaniuk RJ Torsello A. The role of androgens in women’s health and wellbeing. Pharmacol Res. 2021;171:105758. 10.1016/j.phrs.2021.105758 34242799

[4] Gravitte A Archibald T Cobble A Kennard B Brown S. Liquid chromatography–mass spectrometry applications for quantification of endogenous sex hormones. Biomed Chromatogr. 2021;35: 10.1002/bmc.5036 33226656

[5] Fanelli F Gambineri A Mezzullo M Vicennati V Pelusi C Pasquali R Revisiting hyper- and hypo-androgenism by tandem mass spectrometry. Rev Endocr Metab Disord. 2013;14:185–205. 10.1007/s11154-013-9243-y 23619762

[6] Kane J Middle J Cawood M. Measurement of serum testosterone in women; what should we do? Ann Clin Biochem. 2007;44:5–15. 10.1258/000456307779595896 17270086

[7] Davison SL Bell R Donath S Montalto JG Davis SR. Androgen Levels in Adult Females: Changes with Age, Menopause, and Oophorectomy. J Clin Endocrinol Metab. 2005;90:3847–53. 10.1210/jc.2005-0212 15827095

[8] Skiba MA Bell RJ Islam RM Handelsman DJ Desai R Davis SR. Androgens During the Reproductive Years: What Is Normal for Women? J Clin Endocrinol Metab. 2019;104:5382–92. 10.1210/jc.2019-01357 31390028

[9] Ozarda Y. Establishing and using reference intervals. Turk J Biochem. 2020;45:1–10. 10.1515/tjb-2017-0299

[10] Clinical and Laboratory Standards Institute (CLSI). Defining, establishing, and verifying reference intervals in the clinical laboratory; Approved guideline - Third Edition. CLSI EP28-A3c. Wayne:CLSI; 2010.

[11] Haeckel R Wosniok W Streichert T. Review of potentials and limitations of indirect approaches for estimating reference limits/intervals of quantitative procedures in laboratory medicine. J Lab Med. 2021;45:35–53. 10.1515/labmed-2020-0131

[12] Jones GRD Haeckel R Loh TP Sikaris K Streichert T Katayev A Indirect methods for reference interval determination – review and recommendations. Clin Chem Lab Med. 2018;57:20–9. 10.1515/cclm-2018-0073 29672266

[13] Sikaris KA. Separating disease and health for indirect reference intervals. J Lab Med. 2021;45:55–68. 10.1515/labmed-2020-0157

[14] Zierk J Arzideh F Kapsner LA Prokosch H-U Metzler M Rauh M. Reference Interval Estimation from Mixed Distributions using Truncation Points and the Kolmogorov-Smirnov Distance (kosmic). Sci Rep. 2020;10:1704. 10.1038/s41598-020-58749-2 32015476 PMC6997422

[15] Ozarda Y Ichihara K Jones G Streichert T Ahmadian R. Comparison of reference intervals derived by direct and indirect methods based on compatible datasets obtained in Turkey. Clin Chim Acta. 2021;520:186–95. 10.1016/j.cca.2021.05.030 34081933

[16] Omuse G Ichihara K Maina D Hoffman M Kagotho E Kanyua A Determination of reference intervals for common chemistry and immunoassay tests for Kenyan adults based on an internationally harmonized protocol and up-to-date statistical methods. PLoS One. 2020;15: 10.1371/journal.pone.0235234 32645006 PMC7347104

[17] Reynders M Anckaert E Schiettecatte J Smitz J. Evaluation of a new automated electrochemiluminescent sex hormone-binding globulin (SHBG) immunoassay. Clin Chem Lab Med. 2005;43:86–9. 10.1515/CCLM.2005.013 15653448

[18] Handelsman DJ Sikaris K Ly LP. Estimating age-specific trends in circulating testosterone and sex hormone-binding globulin in males and females across the lifespan. Ann Clin Biochem. 2016;53:377–84. 10.1177/0004563215610589 26438522

[19] Imran HJ Dhaher SA Mansour AA. Testosterone or Dehydroepiandrosterone Sulfate as a Biomarker for Hirsutism in Women with Polycystic Ovary Syndrome. Biomed Pharmacol J. 2020;13:1815–23. 10.13005/bpj/2056

[20] Bae YJ Zeidler R Baber R Vogel M Wirkner K Loeffler M Reference intervals of nine steroid hormones over the life-span analyzed by LC-MS/MS: Effect of age, gender, puberty, and oral contraceptives. J Steroid Biochem Mol Biol. 2019;193:105409. 10.1016/j.jsbmb.2019.105409 31201927

[21] Eisenhofer G Peitzsch M Kaden D Langton K Pamporaki C Masjkur J Reference intervals for plasma concentrations of adrenal steroids measured by LC-MS/MS: Impact of gender, age, oral contraceptives, body mass index and blood pressure status. Clin Chim Acta. 2017;470:115–24. 10.1016/j.cca.2017.05.002 28479316 PMC5504266

[22] Haring R Hannemann A John U Radke D Nauck M Wallaschofski H Age-Specific Reference Ranges for Serum Testosterone and Androstenedione Concentrations in Women Measured by Liquid Chromatography-Tandem Mass Spectrometry. J Clin Endocrinol Metab. 2012;97:408–15. 10.1210/jc.2011-2134 22162468

[23] Kushnir MM Blamires T Rockwood AL Roberts WL Yue B Erdogan E Liquid Chromatography–Tandem Mass Spectrometry Assay for Androstenedione, Dehydroepiandrosterone, and Testosterone with Pediatric and Adult Reference Intervals. Clin Chem. 2010;56:1138–47. 10.1373/clinchem.2010.143222 20489135

[24] Büttler RM Kruit A Blankenstein MA Heijboer AC. Measurement of dehydroepiandrosterone sulphate (DHEAS): A comparison of Isotope-Dilution Liquid Chromatography Tandem Mass Spectrometry (ID-LC-MS/MS) and seven currently available immunoassays. Clin Chim Acta. 2013;424:22–6. 10.1016/j.cca.2013.04.028 23665079

[25] Obermayer-Pietsch B de Ramon M Reichmuth C Bendig G Hutzler S Taibon J Multicenter Evaluation of a New, Fully Automated Androstenedione Electrochemiluminescence Immunoassay: Precision Analysis, Method Comparison, and Determination of Reference Ranges. J Appl Lab Med. 2022;7:503–14. 10.1093/jalm/jfab107 34662384

[26] Wei R Bowers K Kroner GM Payto D Colón-Franco JM. The Androstenedione Roche Elecsys immunoassay has superior comparability to the LC-MS/MS assay than the Siemens Immulite immunoassay. Pract Lab Med. 2022;31:e00279. 10.1016/j.plabm.2022.e00279 35620064 PMC9127399

[27] Fanelli F Gambineri A Belluomo I Repaci A Di Lallo VD Di Dalmazi G Androgen Profiling by Liquid Chromatography–Tandem Mass Spectrometry (LC-MS/MS) in Healthy Normal-Weight Ovulatory and Anovulatory Late Adolescent and Young Women. J Clin Endocrinol Metab. 2013;98:3058–67. 10.1210/jc.2013-1381 23780369

[28] Pesant M-H Desmarais G Fink GD Baillargeon J-P. Reference ranges for total and calculated free and bioavailable testosterone in a young healthy women population with normal menstrual cycles or using oral contraception. Clin Biochem. 2012;45:148–50. 10.1016/j.clinbiochem.2011.10.001 22019954

